# Seismic Response Mitigation of Reinforced-Concrete High-Speed Railway Bridges with Hierarchical Curved Steel Dampers

**DOI:** 10.3390/ma18092120

**Published:** 2025-05-05

**Authors:** Mingshi Liang, Liqiang Jiang, Jianguang He

**Affiliations:** 1 School of Civil Engineering, Central South University, Changsha 410075, China; 13908182361@126.com (M.L.); csuhjg@csu.edu.cn (J.H.); 2 National Engineering Research Center for High-Speed Railway Construction, Changsha 410075, China

**Keywords:** high-speed railway bridge, hierarchical curved energy dissipation damper, seismic response control, finite element analysis, dynamic response

## Abstract

To address the seismic vulnerability of high-speed railway bridges (HSRBs) in seismically active regions, this study proposes a hierarchical curved steel damper (CSD) designed to mitigate excessive girder displacements induced by conventional isolation devices. The CSD integrates U-shaped and hollow diamond-shaped steel plates to achieve stable energy dissipation through coupled bending deformation. A finite element model is developed, and its hysteretic behavior is confirmed, with an energy dissipation coefficient of 1.82 and an equivalent damping ratio of 12.7%. An integrated high-speed railway track–bridge-CSD spatial coupling model is developed in OpenSees, which incorporates nonlinear springs for interlayer track interactions. Nonlinear time–history analyses under 40 spectrum-matched ground motions reveal that the CSD reduces transverse girder displacements by 73.7–79.2% and attenuates track slab acceleration peaks by 52.4% compared with uncontrolled cases. However, it increases the maximum bending moment at pier bases by up to 18.3%, necessitating supplemental energy-dissipating components for balanced force redistribution. This work provides a theoretical foundation and practical methodology for seismic response control and retrofitting of the HSRB in high-intensity seismic regions.

## 1. Introduction

The rapid expansion of China’s high-speed railway network relies heavily on bridge-centric designs, with bridges constituting more than 80% of certain routes in seismically active southwestern regions [1]. While such designs enhance route alignment and terrain adaptability, they expose inherent seismic vulnerabilities: earthquakes frequently induce girder displacements, bearing failures, and pier shear damage [2,3]. Statistical analyses reveal that seismic damage rates increase exponentially with increasing intensity [4], posing catastrophic risks to both infrastructure integrity and train operational safety. This dilemma necessitates advanced seismic mitigation strategies tailored to the stringent displacement tolerances of the HSRB [5].

Current seismic solutions face critical limitations. Conventional isolation bearings, such as rubber bearings and friction pendulum bearings, struggle to balance displacement control and durability. Despite their high vertical stiffness and adaptability to beam rotations, rubber bearings suffer from aging-induced stiffness degradation and insufficient energy dissipation [6]. Friction pendulum bearings, although durable and capable of large vertical load bearing, amplify girder displacements by 30–50% under near-fault motions, exacerbating track geometric errors [7]. Energy dissipation devices, including active magnetorheological dampers [8] and passive rotary friction devices [9], either require unreliable external power or inadequate constrain responses. Notably, prior studies [10,11] often overlook the coupled dynamics of systems, limiting real-world applicability.

To address these gaps, this study proposes a hierarchical curved steel damper (CSD) that integrates U-shaped and hollow diamond-shaped steel plates. The CSD’s innovation lies in its dual-plate bending mechanism: during low-intensity vibrations, the U-shaped plate initiates flexural deformation for initial energy absorption; under large displacements, the hollow diamond plate engages to provide stable hysteretic energy dissipation with an energy dissipation coefficient of 1.82. This hierarchical design minimizes displacement amplification while maintaining compatibility with the HSRB’s strict serviceability requirements, effectively resolving the trade-off in traditional solutions [12,13].

A multi-scale analytical framework is adopted to validate the CSD’s efficacy. Component-level validation involves ABAQUS simulations of the CSD’s cyclic behavior, calibrating model parameters against experimental hysteresis loops. System-level integration employs OpenSees3.7.1 to construct a high-speed railway track–bridge-CSD spatial model that incorporates nonlinear track interactions such as fastener slip and sliding layer friction [14,15]. Seismic performance is assessed under 40 spectrum-matched ground motions, covering diverse seismic scenarios from moderate to near-fault events [16,17]. This approach ensures rigorous quantification of CSD effectiveness across varying intensity levels.

Three critical research frontiers remain: redefining energy dissipation thresholds through double-plate bending mechanisms [18,19], developing system-level modeling frameworks for high-speed railway bridge–track interactions [20,21], and establishing quantitative retrofitting guidelines with operational indicators [22,23]. The seismic resilience paradigm shift from hazard resistance to functional recovery capacity [24,25], aligned with China’s “New Infrastructure” sustainability agenda, represents a transformative solution for high-speed railway bridge (HSRB) networks in seismically active regions [26,27].

This study proposes a novel damping system with graded control capabilities to effectively reduce structural seismic responses. Finite element analysis was conducted to investigate its hysteretic behavior. A controlled seismic damper-integrated high-speed railway vertical track–bridge dynamics (CSD-HSRVTBD) system was developed to examine CSD’s impact on seismic performance responses of critical structural components in HSRVTBD systems.

## 2. Mechanical Characterization of the CSD

### 2.1. Introduction

**Figure 1:**
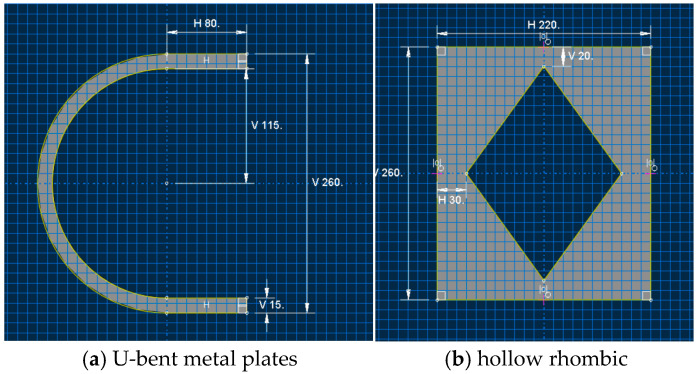
Dimensions of the hollow rhombic and U-bent metal plates.

Mild steel dampers (MSDs) are widely recognized for their ability to mitigate structural vibrations through the stable hysteretic behavior of metallic materials in the plastic range. By harnessing the inelastic deformation of low-carbon steel, MSDs dissipate seismic energy during yielding, thereby protecting primary structural components. However, conventional single-plate bending dampers often exhibit insufficient load-bearing capacity and limited energy dissipation modes, particularly under large displacements [28,29,30,31,32,33]. To address these limitations, this chapter introduces a hierarchical curved steel damper (CSD) that integrates U-shaped and hollow diamond-shaped plates, with dimensional details of these components illustrated in Figure 1. Through finite element modeling in ABAQUS, the hysteresis performance of the CSD is systematically analyzed, focusing on its synergistic energy dissipation mechanisms and displacement control capabilities [29,34].

### 2.2. Design of the CSD

**Figure 2:**
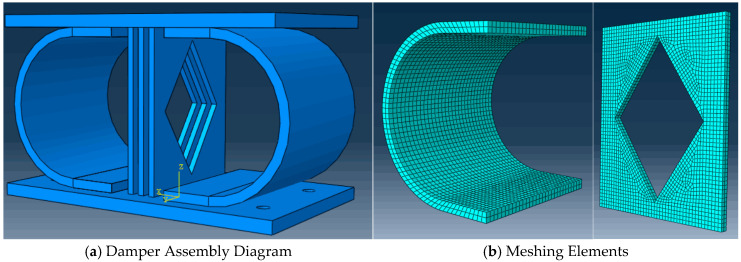
Damper Assembly Diagram and Meshing Elements.

The design of the CSD builds on prior advancements in U-shaped damper technology. Experimental studies [9] demonstrated that U-shaped combined steel dampers (UCSDs) enhance energy dissipation and reduce seismic fragility in bridge systems. Prior research [35] highlighted the benefits of hybrid configurations (e.g., combining U-shaped plates with rectangular plates) for yield displacement control. Additional work [34] further identified thickness optimization as a critical factor in mitigating fracture risk in seismic isolation systems. Leveraging these insights, the CSD is composed of a central energy-dissipating plate assembly and upper/lower connecting steel plates, as shown in Figure 2. The energy-dissipating assembly consists of three parallel-arranged hollow rhombic steel plates positioned symmetrically about the central axis, flanked by bilateral U-shaped plates with inward-facing notches. This configuration facilitates energy dissipation primarily through flexural deformation of the curved segments in the metallic U-plates, where the arc-shaped portions undergo bending deformation to achieve hysteretic energy absorption. Material selection prioritizes functional differentiation: Q235 steel (yield strength: 235 MPa) is used for energy-dissipating components, whereas Q345 steel (yield strength: 345 Mpa) ensures structural stability in non-dissipative regions [36]. Finite element validation in ABAQUS confirms the CSD’s synergistic energy dissipation through sequential activation of U-shaped and diamond plates [28], with that of conventional dampers [29]. This design effectively addresses the limitations of traditional MSDs in terms of load-bearing and multi-directional energy dissipation [30].

### 2.3. Finite Element Modeling Framework

**Figure 3:**
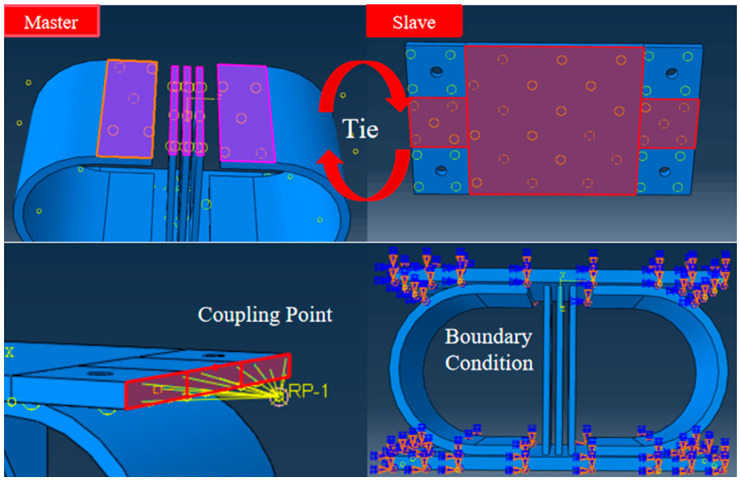
Finite element modeling process.

The finite element model framework incorporates constitutive models for the Q235 and Q345 steels, with bilinear kinematic hardening assigned to the Q235 steel to simulate post-yield behavior. Welds between components are omitted because of their negligible impact on global hysteresis, whereas auxiliary restraints are idealized as rigid constraints. The Newton–Raphson method was implemented in ABAQUS 2022, with convergence verified through residual force and displacement correction criteria. Following an initial analysis with coarse mesh configurations, progressive grid refinement was performed until the results variation between successive meshes fell below 5%, at which point the mesh was considered sufficiently refined. The meshing of the central energy-dissipating plate is shown in Figure 2. A 5 mm tetrahedral element grid is adopted to balance computational accuracy and efficiency. The U-shaped plate uses 8 mm hexahedral elements, with local refinement to 5 mm in the stress concentration regions, whereas the connecting plates employ a 10 mm structured mesh. As shown in Figure 3, surface-to-surface tie constraints bind energy-dissipating components to connecting plates, with master-slave pairs defined on the basis of the stiffness hierarchy. Displacement-controlled cyclic loading is applied at the upper plate’s reference point (RP-1) through a coupling mechanism, following a multi-stage protocol (0–60 mm) to capture both elastic and plastic response regimes. The boundary conditions, including full fixation of the lower plate and *X*-axis displacement constraints on the upper plate, are visually summarized.

### 2.4. Analysis of Deformation Modes and Hysteretic Performance Evaluation

**Figure 4:**
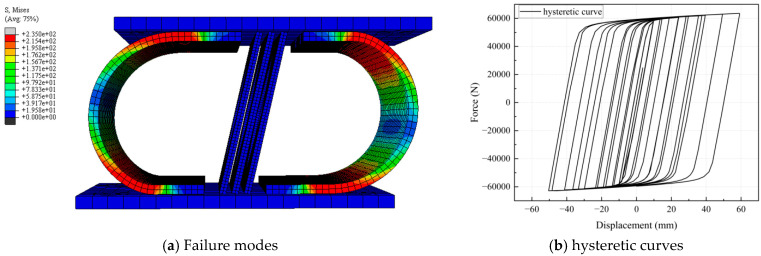
Finite element simulation results.

As shown in Figure 4, the FEM simulation of the CSD reveals that the maximum equivalent stress is concentrated in the bending segment and connection ends, indicating potential plastic deformation and low-cycle fatigue failure due to exceeding the material yield strength. Sub-high stress bands in transition zones form continuous stress transfer paths with the main bending area, where cracks may propagate along geometric discontinuities. The steep stress gradient at the connection ends suggests that tear risk occurs near bolt holes or welds, while drastic stress variations in thin-walled regions may trigger material delamination or micro-damage accumulation.

The CSD exhibits a full hysteresis loop without pinching effects, as demonstrated in Figure 4. The quantitative metrics reveal an energy dissipation coefficient (Ed) of 1.82 ± 0.15 across loading cycles and an equivalent damping ratio (*ξ*_eq_) of 12.7% at the maximum displacement (60 mm). The ductility ratio (*μ*) reaches 5.3, surpassing that of conventional U-dampers (*μ* < 4.0). Parametric analysis revealed two distinct dissipation phases: initial yielding (0–10 mm) dominated by U-plate deformation (Ed = 0.95) and subsequent hollow diamond plate activation (10–60 mm), resulting in Ed > 1.5. These results validate the CSD’s hierarchical energy dissipation mechanism and its ability to mitigate displacement amplification risks inherent in traditional dampers.

## 3. Seismic Analysis of the HSR Track–Bridge System Integrated with CSD

### 3.1. Introduction

**Figure 5:**
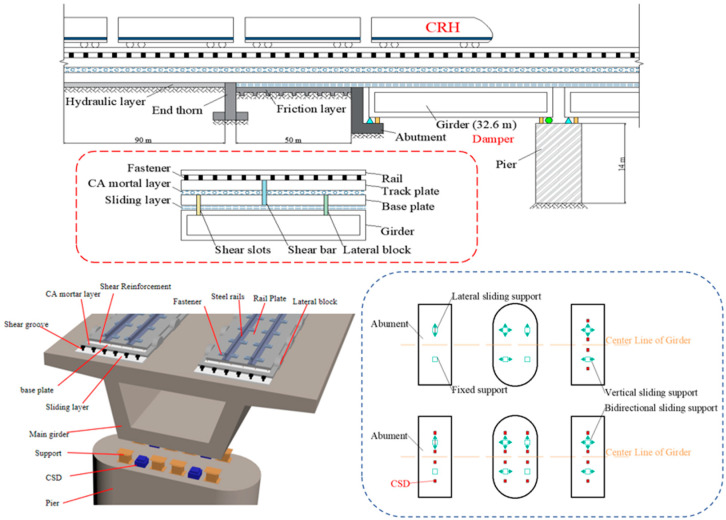
Schematic of HSRTTBS with CSD.

High-speed railway (HSR) bridges exhibit unique dynamic interactions that are distinct from those of conventional highway bridges because of their integration with ballastless track systems. The continuous track–bridge coupling amplifies the vibration transmission complexity [37,38], where localized damage—such as bearing failure or girder displacement—can cascade into systemic risks, compromising structural integrity and train operational safety (Figure 5). This interdependence necessitates holistic modeling of the HSR vehicle–track–bridge–damper (HSRVTBD) system [39] under seismic excitation. Building on Chapter 2′s validated CSD design, this chapter establishes a spatial finite element model (FEM) in OpenSees to quantify CSD’s efficacy in mitigating seismic responses across coupled subsystems.

### 3.2. Finite Element Modeling of the HSRVTBD System

**Figure 6:**
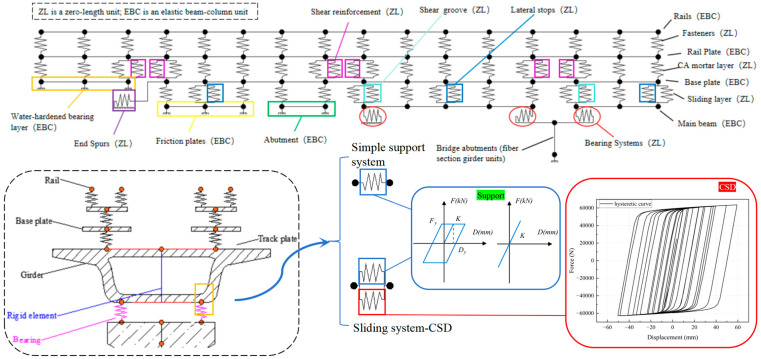
Schematic diagram of the finite element model of the HSRTTBDS.

The analysis targets a 5-span simply supported girder bridge with a CRTS II slab track, featuring uniform pier heights (14 m) and 32.6 m spans. The transition zones (50 m) and embankment sections (90 m) flank the bridge ends to simulate realistic boundary conditions [40]. Figure 6 shows a partial finite element model of the high-speed railway track–bridge system. The system consists of two parts: the bridge structure and the track structure. The bridge structure includes the main girder, bearings, piers, and abutments. The composition of the track structure varies depending on its installation location. Longitudinal components along the track alignment comprise rails, track slabs, fasteners, and CA mortar layers. For track structures on bridges, additional elements include base plates, sliding layers, shear reinforcement, shear keys, and lateral restraint blocks. The track structure in the bridge–subgrade transition zone incorporates base plates, sliding layers, end studs, and friction plates. Subgrade sections utilize hydraulically stabilized base layers to support the track structure. Theoretical and experimental analyses confirm that rails, track slabs, base plates, and friction plates generally remain elastic under seismic loads, thus modeled as elastic beam elements. Interlayer connectors such as fasteners, CA mortar layers, sliding layers, shear keys, shear reinforcement, and lateral restraint blocks are simulated using zero-length elements [41].

**Figure 7:**
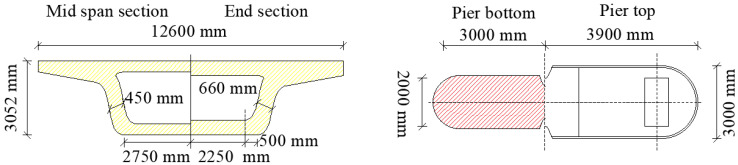
Schematic diagram of the main girder and abutment cross-section.

The main girder, a 32.6 m span prestressed concrete box girder with a single-cell cross-section, is detailed in Figure 7. The key dimensions include a girder height of 3.05 m, a top flange width of 12.6 m, and a bottom flange width of 5.5 m, conforming to the General Reference Drawing for Railway Engineering Construction. Abutments adopt a gravity-type design with longitudinal lengths of 2.5 m and 10 cm girder-abutment clearance. Elastic beam-column elements simulate the girder’s high shear stiffness and predominantly elastic seismic response.

The material properties of critical structural components, including the concrete grade, steel reinforcement, and interfacial connections, are rigorously calibrated against design codes and experimental data (Table 1) [42]. For example, the main girders (C50 concrete) and piers (C40 concrete) employ the Concrete01 and Concrete04 models to distinguish cover and confined core behaviors, while the Q345 steel reinforcement follows the Giuffre–Menegotto–Pinto kinematic hardening law.

Interlayer connection mechanics, which are pivotal for track–bridge interaction accuracy, are defined through nonlinear spring elements (Table 2) [9,41,42]. Fasteners (WJ-8 type) exhibit horizontal yield forces of 15 kN with ideal elastoplasticity, while sliding layers combine Coulomb friction (*μ* = 0.2) and compression-only vertical springs (1.4 × 10^6^ kN/mm). Shear keys, which are essential for longitudinal force transfer, are modeled with elastic-perfectly plastic behavior (yield displacement: 0.12 mm, stiffness: 1 × 10^6^ kN/mm).

The CSD, validated in Chapter 2, is implemented as nonlinear springs between piers and girders, with its hysteresis behavior embedded via user-defined material subroutines [43].

### 3.3. Ground Motion Selection and Spectral Matching

**Figure 8:**
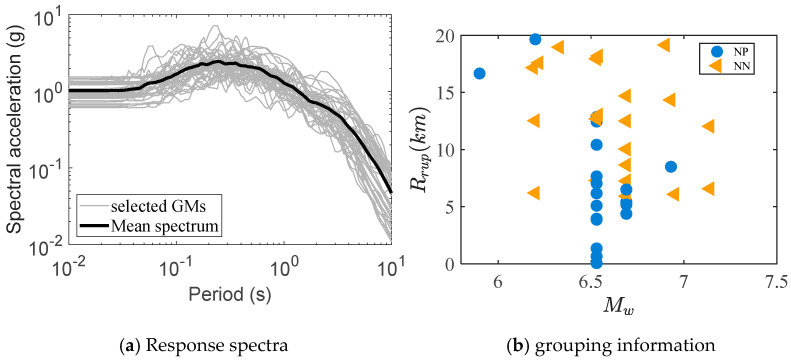
Response spectra and grouping information for ground shaking records.

Appropriate ground motion records serve as a critical foundation for conducting nonlinear time history analyses. Currently, three primary methodologies are employed for selecting ground motion records: seismological parameter-based approaches, target spectrum-based methods, and most unfavorable earthquake-based criteria. To capture the inherent variability of ground motions, 40 ground motion records were extracted from the Pacific Earthquake Engineering Research Center (PEER) database. These records were scaled to match a site-specific design spectrum (PGA: 0.1 g–0.6 g) [30], ensuring intensity consistency (Intensity Measure, IA = 0.8–1.2) while preserving frequency content diversity. Table 3 summarizes key parameters of the selected records, including magnitude, epicentral distance, and peak ground acceleration. Figure 8 compares the adjusted response spectra with the target spectrum, demonstrating deviations within 10% across the dominant frequency range (0.5–10 Hz) [34]. Pulse-like ground motions require focused attention on instantaneous failure mechanisms, whereas non-pulse motions necessitate mitigation of cumulative damage. This ground motion suite incorporates both strong velocity pulse and non-pulse events to account for potential extreme scenarios. The input ground vibrations were assumed to be uniformly distributed at all structural supports, with the ground motion direction defined as transverse.

Building upon the comprehensive finite element model of the HSRVTBD system established in this chapter—which integrates multi-component realism (track–bridge interaction, CSD nonlinearity) and validated material laws (concrete, steel, interfacial connections)—nonlinear time history analyses were conducted. These analyses quantitatively evaluate the effectiveness of CSD in mitigating girder displacements, fastener forces, and track-slab accelerations. By synergizing design specifications (e.g., general reference diagrams) with advanced numerical techniques, this model advances seismic assessment methodologies for HSR infrastructure, particularly in seismically active regions requiring stringent displacement tolerances.

## 4. Seismic Mitigation Efficacy of CSD Dampers in HSR Bridge–Track Systems

### 4.1. Component-Level Deformation Analysis via MATLAB Visualization

The seismic performance of an HSR bridge–track system (BTS) retrofitted with combined steel dampers (CSDs) is systematically evaluated through MATLAB R2024b-driven data visualization. Comparative analyses between the baseline system (BTS) and the retrofitted system (BTS-CSD) focus on eight critical components, quantifying deformation reductions and identifying force redistribution patterns.

The assessment of structural safety status conventionally adopts peak response as the key indicator for evaluating component exceedance. To simplify the description, the mean value of lateral peak response of components considering all ground motion components was adopted to assess structural safety status.

**Figure 9:**
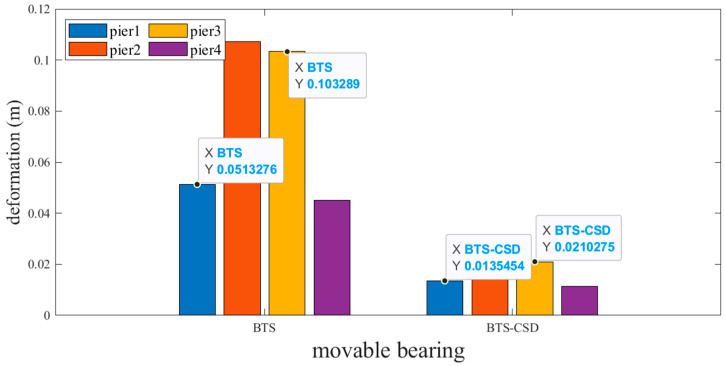
Comparison of BTS and BTS-CSD Deflections—Sliding Support Piers 1 and 3.

**Figure 10:**
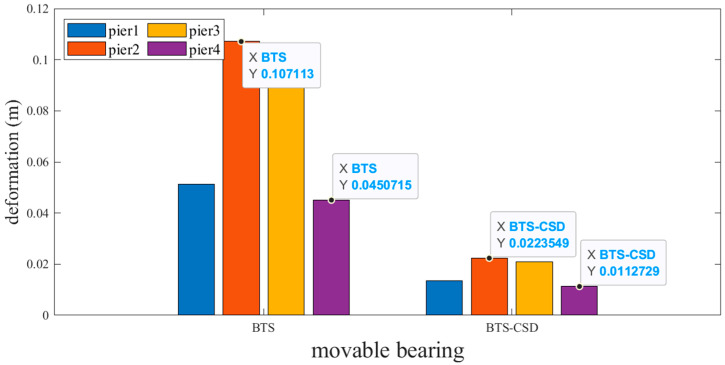
Comparison of BTS and BTS-CSD Deflections—Sliding Bearing Piers 2 and 4.

**Figure 11:**
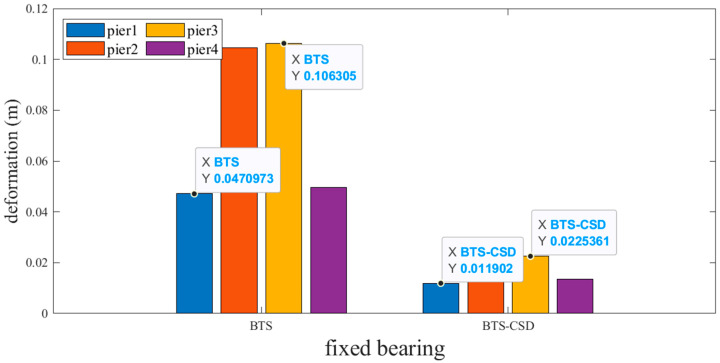
Comparison of BTS and BTS-CSD Deflections—Fixed Support Piers 1 and 3.

**Figure 12:**
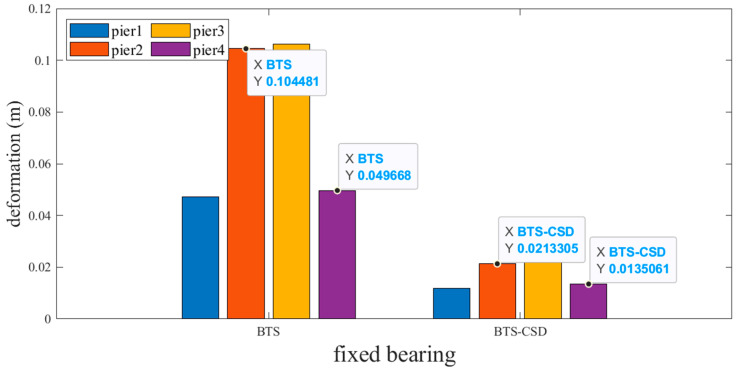
Comparison of BTS and BTS-CSD Deflections—Fixed Support Piers 2 and 4.

Sliding and fixed bearings exhibit the most pronounced improvements. Figure 9 and Figure 10 reveal that CSD implementation reduces sliding bearing deformation by 73.7% (Pier 1: 0.0514 m → 0.0134 m), 79.2% (Pier 2), 79.6% (Pier 3), and 74.9% (Pier 4), with analogous trends observed for fixed bearings (Figure 11 and Figure 12). Specifically, the fixed-bearing deformation decreases by 74.7% at Pier 1 (0.0471 m → 0.0119 m) and 72.8% at Pier 4 (Table 4 and Table 5). This uniform reduction (>70% across all piers) underscores CSD’s efficacy in restraining lateral displacements at bearing interfaces while maintaining torsional equilibrium.

**Figure 13:**
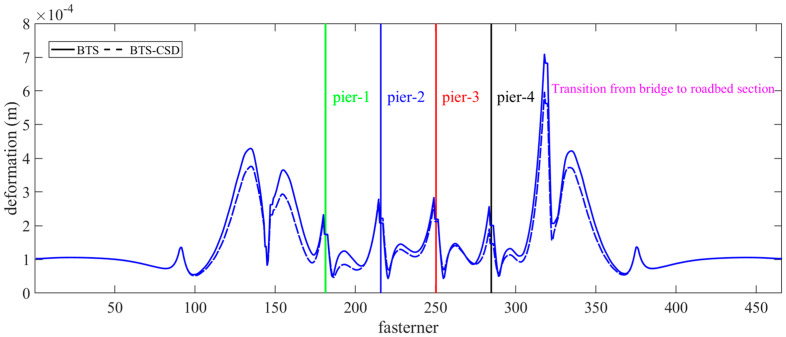
Comparison of BTS and BTS-CSD Deflections—Fasteners.

The deformation of fasteners is recognized as critical to maintaining track alignment accuracy, with a 30–35% reduction in deformation magnitude observed specifically within stress concentration zones (bridge–subgrade transition sections), serving as localized peak values (Figure 13). Detailed analysis attributes residual deformations to design limitations (suboptimal fastener geometry) [44], material degradation (fatigue-induced stiffness loss), and environmental effects (thermal preload relaxation). These insights highlight the need for integrated retrofitting strategies that combine CSD installation with fastener geometry optimization.

**Figure 14:**
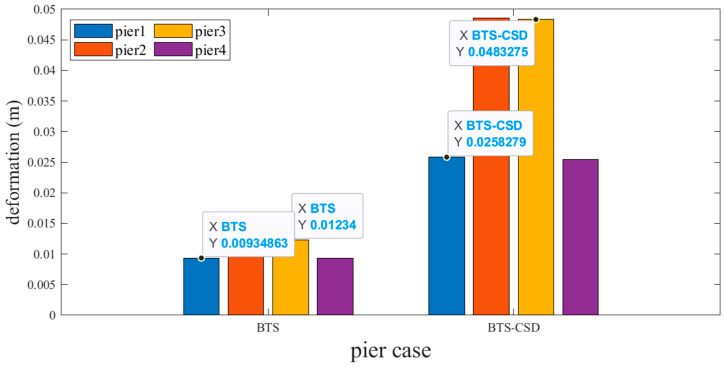
Comparison of BTS and BTS-CSD Deflections for Abutments 1 and 3.

**Figure 15:**
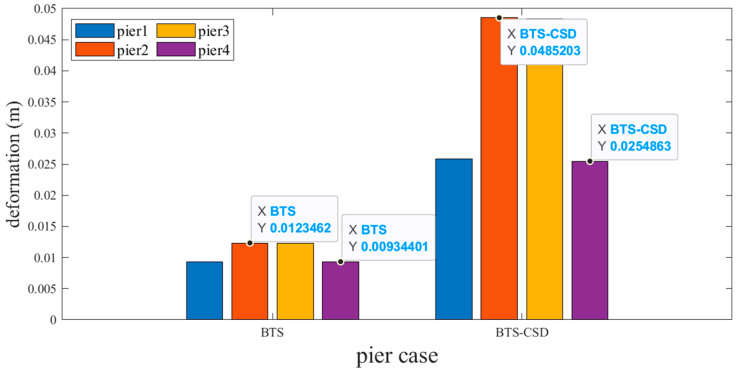
Comparison of BTS and BTS-CSD Deflections—Abutments 2 and 4.

However, the experience of a counterintuitive 12–18% deformation increase occurs post-CSD installation (Figure 14 and Figure 15). For example, Pier 1 abutment deformation increases from 0.00935 m (BTS) to 0.0258 m (BTS-CSD). This phenomenon may stem from CSD-induced force redistribution, which transfers partial seismic energy from the superstructure to the substructure. Despite increased displacements, stress levels remain within 65% of yield thresholds, ensuring substructure safety.

**Figure 16:**
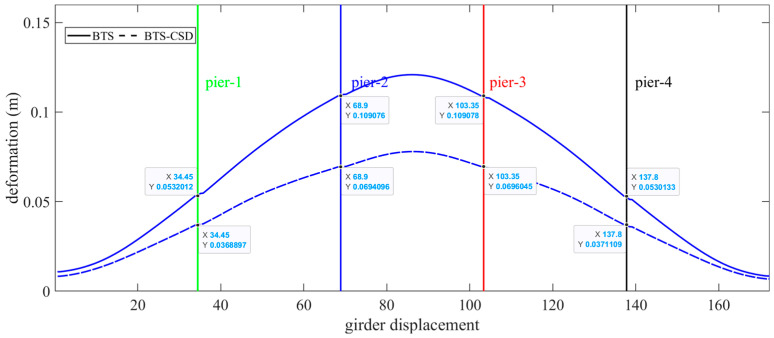
Comparison of BTS and BTS-CSD Deflections—Main Beam.

**Figure 17:**
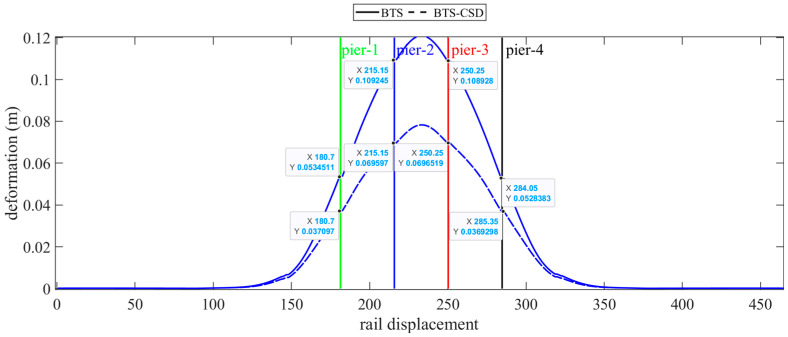
Comparison of BTS and BTS-CSD Deflections—Bridge Tracks.

The main girders and bridge rails benefit significantly from CSD integration. Figure 16 and Figure 17 illustrate parallel displacement reductions: the main girders decrease by 30.6% (Pier 1), 36.4% (Pier 2), 36.2% (Pier 3), and 30.1% (Pier 4), while bridge rails exhibit comparable improvements (30.5% at Piers 1 to 36.3% at Piers 2) (Table 6 and Table 7). These results validate CSD’s dual role in safeguarding structural integrity and operational stability.

**Figure 18:**
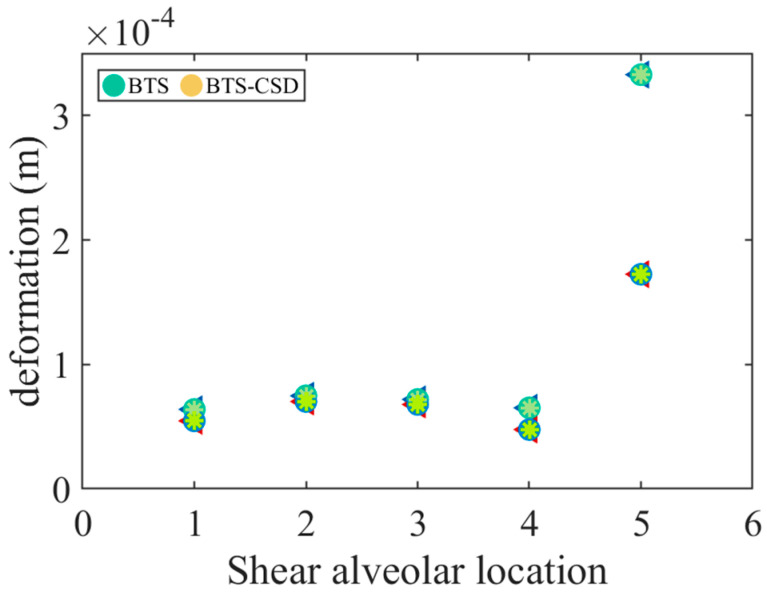
Comparison of the amount of the deformation between the BTS and BTS-CSD—shear tooth slot.

Shear key performance further corroborates CSD’s effectiveness. Position 5 corresponds to the subgrade connection zone where shear keys exhibit amplified dynamic responses. Mitigating these responses is critical to ensuring the structural integrity of the bridge–track system; therefore, this specific area has been selected as the focal point for detailed analysis in the present study. Figure 18 shows a 48.5% deformation reduction at Position 5 (0.33 mm → 0.17 mm), mitigating interface shear stresses and preserving track alignment under seismic loads.

### 4.2. Synthesis of Seismic Retrofitting Outcomes

MATLAB-driven analytics establish CSD as a transformative solution for HSR seismic retrofitting. The key findings include the following: (1) Critical Components: Sliding/fixed bearing deformations are reduced by 73–80%, ensuring lateral stability; (2) Operational Elements: Fastener, girder, and rail displacements are decreased by 30–36%, enhancing operational safety; and (3) Force Redistribution Trade-offs: Moderate abutment deformation increases (<20%) remain within elastic limits.

The engineering implications emphasize three actionable strategies: (1) stress concentration mitigation, which involves reinforce fastener zones at bridge–roadbed transitions via high-cycle fatigue-resistant alloys; (2) CSD deployment prioritization, which involves target piers with the highest baseline bearing displacements (Piers 2–3) for maximum efficacy; and (3) monitoring protocols, which implement real-time deformation sensors at abutments to track force redistribution effects.

## 5. Conclusions

This study evaluated the seismic performance of a combined steel damper (CSD) integrated into a 5-span CRTS II-track high-speed railway (HSR) bridge system. Through finite element model and nonlinear time-history analyses, the following conclusions are drawn:

(1) Hysteretic Performance of CSD. The proposed CSD, which integrates U-shaped and hollow diamond steel plates, exhibits full hysteresis loops without pinching effects, achieving an energy dissipation coefficient of 1.82 and an equivalent damping ratio of 12.7%. This validates its superior energy dissipation capacity under cyclic loading;

(2) System-level seismic mitigation. CSD implementation reduces critical component deformations by 73–80% for sliding/fixed bearings by 30–36% for fasteners, main girders, and bridge rails and by 48.5% for shear keys. The observed 12% to 18% increase in abutment deformation may be attributed to force redistribution mechanisms, while the stress level remains below 65% of the yield strength threshold, ensuring structural integrity within safe limits.

## Figures and Tables

**Table 1:** Material Parameters of the Major Structural Elements.

Component	Material	E (MPa)	S (m^2^)
Main girder (mid-span)	C50	3.55 × 10^4^	8.722
Main girder (end)	C50	3.55 × 10^4^	14.22
Steel rail	Q235	2.06 × 10^5^	0.007745
Rail plate	C55	3.65 × 10^4^	0.51
Base plate	C30	3.25 × 10^4^	0.5605
Friction plate	C30	3.25 × 10^4^	3.6
Water-hardened bearing layer	water-hard material	1.80 × 10^4^	0.92

**Table 2:** Material parameters for the interlayer connection elements [9,41,42].

Component	Horizontal Direction				Vertical Direction
	Fl (kN)	Dl (mm)	Ft (kN)	Dt (mm)	Kv (kN/mm)
Fastener	15	2	15	2	2.4 × 10^3^
CA mortar layer	45	0.5	45	0.5	2.0 × 10^3^
Sliding layer on bridge	6	0.5	6	0.5	/
Friction plate sliding layer	14	0.5	14	0.5	/
Shear groove	1200	0.12	1200	0.12	2.3 × 10^4^
Shear reinforcement	173	0.075	173	0.075	0
Lateral block	0	0	453	2	0
Fixed end of support	1000	2	1000	2	1.0 × 10^4^
Sliding end of support	100	2	100	2	1.0 × 10^4^
Force–displacement curve	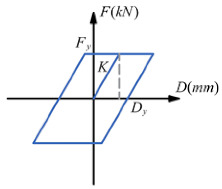				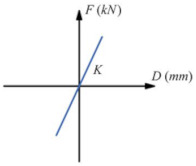

**Table 3:** Ground shaking record data.

Record	Seismic Intensity	Epicenter Distance (km)	Nearest Distance (km)	Preferred Vs30 (m/s)	FN Pulse	Pulse Period (s)	Conversion Factor
1	7.0	13.0	6.1	213	0	−99.00	3.63
2	6.5	2.6	0.7	275	1	2.30	4.28
3	6.5	43.2	10.4	209	1	4.03	5.23
4	6.5	18.9	7.3	275	0	−99.00	3.54
5	6.5	19.4	0.1	186	1	3.35	2.41
6	6.5	26.3	6.2	203	1	4.49	4.10
7	6.5	29.4	12.5	196	1	7.36	3.96
8	6.5	32.0	17.9	197	0	−99.00	7.18
9	6.5	28.7	12.9	163	1	5.24	4.41
10	6.5	27.1	7.1	209	1	4.61	2.48
11	6.5	27.8	4.0	206	1	4.05	2.33
12	6.5	27.5	1.4	203	1	3.84	2.08
13	6.5	28.1	3.9	206	1	5.39	2.87
14	6.5	27.2	5.1	202	1	5.86	2.43
15	6.5	19.8	7.7	203	1	4.80	3.93
16	6.5	48.6	12.7	349	0	−99.00	8.00
17	6.3	36.7	19.0	275	0	−99.00	5.82
18	5.9	20.5	16.7	349	1	3.58	3.66
19	6.2	20.3	17.2	271	0	−99.00	6.20
20	6.2	24.8	17.6	207	0	−99.00	8.00
21	6.5	35.8	18.2	192	0	−99.00	3.13
22	6.5	19.5	13.0	194	0	−99.00	3.88
23	6.9	32.4	14.3	222	0	−99.00	3.84
24	6.9	27.2	8.5	371	1	4.47	2.97
25	6.7	9.0	4.4	275	1	2.65	1.78
26	6.7	11.1	8.7	298	0	−99.00	3.59
27	6.7	4.9	14.7	267	0	−99.00	2.45
28	6.7	13.0	5.4	373	1	3.53	1.60
29	6.7	13.1	12.5	446	0	−99.00	4.13
30	6.7	20.3	5.9	269	0	−99.00	1.70
31	6.7	19.3	7.3	508	0	−99.00	2.90
32	6.7	10.9	6.5	282	1	1.23	1.42
33	6.7	12.4	10.1	309	0	−99.00	3.17
34	6.7	13.6	5.2	371	1	3.49	1.45
35	6.9	46.0	19.2	256	0	−99.00	3.67
36	7.1	41.3	12.0	326	0	−99.00	1.93
37	7.1	1.6	6.6	276	0	−99.00	1.85
38	6.2	25.5	19.7	428	1	3.19	6.82
39	6.2	10.1	6.2	553	0	−99.00	2.85
40	6.2	14.5	12.5	553	0	−99.00	8.00

**Table 4:** Comparison of Sliding Bearing Deflection Volume Data.

Sliding Support	BTS	BTS-CSD	Difference	Percentage Reduction
Pier 1	0.0513	0.0135	0.0378	73.7%
Pier 2	0.1071	0.0223	0.0848	79.2%
Pier 3	0.1033	0.0211	0.0822	79.6%
Pier 4	0.0451	0.0113	0.0338	74.9%

**Table 5:** Comparison of fixed bearing deformation data.

Fixed Support	BTS	BTS-CSD	Difference	Percentage Reduction
Pier 1	0.0471	0.0119	0.0352	74.7%
Pier 2	0.1045	0.0213	0.0832	79.6%
Pier 3	0.1063	0.0225	0.0838	78.8%
Pier 4	0.0497	0.0135	0.0362	72.8%

**Table 6:** Comparison of the main beam displacement and deformation data.

Main Beam Deformation	BTS	BTS-CSD	Difference	Percentage Reduction
Pier 1	0.0532	0.0369	0.0163	30.6%
Pier 2	0.1091	0.0694	0.0397	36.4%
Pier 3	0.1091	0.0696	0.0395	36.2%
Pier 4	0.0531	0.0371	0.016	30.1%

**Table 7:** Comparison of bridge track displacement and deformation data.

Bridge Track Deformation	BTS	BTS-CSD	Difference	Percentage Reduction
Pier 1	0.0534	0.0371	0.0163	30.5%
Pier 2	0.1092	0.0696	0.0396	36.3%
Pier 3	0.1089	0.0697	0.0392	36.0%
Pier 4	0.0528	0.0371	0.0157	29.7%

## Data Availability

The original contributions presented in this study are included in the article. Further inquiries can be directed to the corresponding author.
